# Hybrid-PET/MRT bei inflammatorischer Kardiomyopathie

**DOI:** 10.1007/s00117-022-01064-w

**Published:** 2022-09-02

**Authors:** Patrick Krumm, Simon Greulich, Christian la Fougère, Konstantin Nikolaou

**Affiliations:** 1 Diagnostische und Interventionelle Radiologie, Universitätsklinikum Tübingen, Hoppe-Seyler-Str. 3, 72076 Tübingen, Deutschland; 2 Innere Medizin III, Kardiologie und Angiologie, Universitätsklinikum Tübingen, Tübingen, Deutschland; 3 Nuklearmedizin und Klinische Molekulare Bildgebung, Universitätsklinikum Tübingen, Tübingen, Deutschland

**Keywords:** Positronen-Emissions-Tomographie, Magnetresonanztomographie, Inflammatorische Kardiomyopathie, Sarkoidose, Myokarditis, PET/MRI, MR-PET, Inflammatory cardiomyopathy, Sarcoidosis, Myocarditis

## Abstract

**Hintergrund:**

Die Myokarditis und die inflammatorische Kardiomyopathie sind aufgrund ihrer unterschiedlichen Auslöser, Phänotypen und Stadien diagnostisch häufig schwer zu diagnostizieren.

**Methodische Innovationen und Probleme:**

Die kardiale Positronen-Emissions-Tomographie/Magnetresonanztomographie (PET/MRT) zeichnet sich neben der myokardialen Gewebecharakterisierung mittels MRT durch den möglichen Nachweis einer aktiven myokardialen Entzündung (Inflammation) mittels PET aus. Die Kombination von MRT und PET ist somit eher synergistisch als rein summativ: Die möglicherweise in der MRT vorhandenen kardialen Veränderungen lassen sich durch die PET in aktive inflammatorische (und somit noch potenziell reversible) Prozesse oder ältere chronische (irreversible) Narben unterscheiden. Die kardiale Sarkoidose mit einem potenziellen Nebeneinander von aktiven und chronischen Veränderungen bietet sich an, um die Stärken einer hybriden PET/MRT zur Geltung bringen zu lassen. Wichtig für eine aussagekräftige kardiale PET ist eine gute Vorbereitung mit Low-Carb-Diät, um eine suffiziente Suppression der myokardialen Glukoseaufnahme zu gewährleisten.

**Empfehlungen:**

Die Diagnostik einer inflammatorischen Herzerkrankung sowie deren Charakterisierung in akut vs. chronische Prozesse gelingt mit der kardialen Hybrid-PET/MRT, wie am Beispiel der kardialen Sarkoidose gezeigt werden konnte.

## Abstract (en)

**Background:**

Diagnostic work-up for myocarditis and inflammatory cardiomyopathy is challenging for its different aetiologies, phenotypes and stages.

**Methodical innovations and challenges:**

Cardiac PET/MRI is an excellent tool for myocardial tissue characterization with MRI as well as for diagnosis of active myocardial inflammation with PET. The combination of MRI and PET is rather synergistic than only summative: myocardial changes detected by MRI can be differentiated with PET to (potentially still reversible) active inflammation vs. chronic and inactive (irreversible) scars.

The strengths of a hybrid PET/MRI can show to advantage in cardiac sarcoidosis with potentially coexistent active and chronic involvement. Good dietary preparation with a low-carb diet is essential to warrant sufficient suppression of myocardial glucose uptake for robust cardiac PET in context of inflammatory cardiomyopathy.

**Recommendations:**

Hybrid cardiac PET/MRI to diagnose inflammatory cardiomyopathy and further differentiation in acute vs. chronic processes is feasible. This could be demonstrated in cardiac involvement of sarcoidosis.

Die Positronen-Emissions-Tomographie (PET) und Magnetresonanztomographie (MRT) sind etablierte Methoden in der modernen kardialen Bildgebung. Bislang werden jedoch nur an wenigen Standorten routinemäßig kardiale PET/MRT-Untersuchungen durchgeführt, wobei die Nachfrage nach diesen Untersuchungen kontinuierlich steigt.

Die Untersuchungen können dabei in Abhängigkeit vom Gerätetyp des Scanners entweder als Hybridverfahren integriert simultan oder alternativ seriell nacheinander durchgeführt werden [1]. Das modernere Verfahren stellt die kardiale Hybrid-PET/MRT-Untersuchung dar, ist sie doch zeitsparender und für Patient:innen komfortabler.

In diesem Übersichtsartikel sollen insbesondere die aktuellen Anwendungsmöglichkeiten der hybriden kardialen Bildgebung mit dem Fokus auf inflammatorische Kardiomyopathie am Beispiel der kardialen Sarkoidose aufgezeigt werden.

## Untersuchungsmodi der Herz-PET

### Inflammation vs. Vitalität

Myokardzellen können sowohl Glukose als auch freie Fettsäuren verstoffwechseln [2]. Für die Darstellung einer myokardialen Entzündung muss folglich ein anderes PET-Untersuchungsprotokoll verwendet werden als für die Darstellung der myokardialen Vitalität. Für die Vitalitätsbildgebung, die überwiegend mit dem Glukoseanalogon [^18^F]2-Fluor-2-desoxy-D-glucose (FDG) durchgeführt wird, ist die Vorbereitung mit einem Glukose-Loading vor Tracerapplikation notwendig, um einen maximalen FDG-Uptake der gesunden Myozyten zu gewährleisten [3]. Somit können myokardiale Narben ohne FDG-Verstoffwechselung von ischämischem aber noch vitalem („hibernating“) Myokard mit erhaltenem Glukosestoffwechsel unterschieden werden. Ferner kann die myokardiale Perfusion in der PET mittels geeigneter Perfusionstracer wie z. B. [^13^N]NH_3_ (Amonia), [^15^O]H_2_0 (Wasser) und [^82^Rb] (Rubidium) untersucht werden [2].

Kardiale PET/MRT-Untersuchungen sind zwar sehr sensitiv in der Vitalitätsdiagnostik [4], jedoch auch sehr aufwendig – insbesondere, wenn zwei verschiedene Tracer für Perfusion und Vitalität genutzt werden [1, 5]. Erschwerend kommt hinzu, dass mit anderen nichtinvasiven Verfahren wie der kardialen Stress-MRT und Perfusions-Single-Photon-Emissions-Computertomographie/Computertomographie (SPECT/CT) breit verfügbare und etablierte Untersuchungen für den Nachweis einer Perfusionsstörung bzw. Narbe vorhanden sind [4–6], die eine rasche Verbreitung der PET/MRT für diese Indikation bislang nicht wesentlich vorangetrieben haben [7].

### PET für inflammatorische Herzerkrankung: Fettstoffwechsel des Myokards

**Figure 1:**
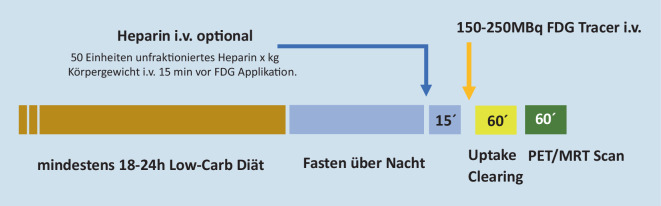

Um die Stoffwechselaktivität von eingewanderten Immunzellen im Myokard [8] messen zu können, muss die physiologische Aufnahme von [^18^F]FDG möglichst vollständig supprimiert werden. Dies kann durch eine konsequente kohlenhydratarme (Low-Carb‑)Diät unter Vermeidung von Kohlenhydraten und Zuckern erreicht werden ([9]; Abb. 1). Für mindestens 18 (besser 24) Stunden vor der geplanten PET-Untersuchung sollen hierfür von Patient:innen nur eiweißreiche und fettreiche Kost (Speck, Ei, fettreicher Fisch, rotes Fleisch und kohlenhydratfreie Gemüse wie Gurken und Tomaten) gegessen werden. Als Getränke sind nur Wasser, ungesüßter Tee und schwarzer Kaffee erlaubt. Unbedingt vermieden werden sollten alle kohlenhydrathaltigen Speisen (Hülsenfrüchte, Erbsen, Mais, Getreide, Nudeln, Käse sowie insbesondere Fertiggerichte und Soßen, und Wurst, welche häufig Zucker enthalten kann) sowie Snacks, Süßigkeiten und Säfte [10].

Unterstützt werden kann die myokardiale FDG-Suppression durch einen Heparinbolus (unfraktioniertes Heparin 50 IE/kg Körpergewicht) 15 min vor Tracerapplikation [10]. Allerdings kann Heparin eine mangelnde diätetische Vorbereitung nicht *retten* oder gar gänzlich ersetzen.

Wichtig ist die entsprechende Kommunikation im Voraus mit den Patient:innen, in der klar die Bedeutung der diätetischen Maßnahmen und die zu erwartende eingeschränkte Aussagekraft der Bilder bei Nichtbeachtung derselben zum Ausdruck gebracht werden sollte. Bei strikter Einhaltung der Diät werden in der Regel gute Ergebnisse der myokardialen Suppression erzielt. Die Dauer bis zur vollständigen Umstellung des Myokards von Glukose- auf kompletten Fettstoffwechsel ist individuell verschieden. Abhängig von der gewohnten Ernährung und Frequenz intermittierenden Fastens können teilweise mit der gleichen diätischen Vorbereitung unterschiedlich gute Ergebnisse erzielt werden. Insbesondere nach Feiertagen wie Weihnachten, mit üblicherweise hochkalorischen Mahlzeiten, wurde eine Koinzidenz mit unzureichender diätetischer Vorbereitung identifiziert. Trotz guter Instruktion sind im klinischen Alltag nur ca. 85 % der Patient:innen optimal vorbereitet, zeigen in den Aufnahmen also eine ausreichende myokardiale Suppression [10–12]. Die individuellen Zusammenhänge und das strikte Einhalten der Diät sind naturgemäß schwierig in kontrollierten Studien zu untersuchen.

Für unsere ambulanten Patient:innen wurde mit der Ernährungsberatung ein Informationsblatt mit konkreten Vorschlägen zu Mahlzeiten und einer ausführlichen Positiv- und Negativliste an Nahrungsmitteln erarbeitet, welches den Patient:innen bei der Terminvergabe übermittelt wird. Für unsere stationären Patient:innen wurde eine Low-Carb-Diät in Form eines bestellbaren Essensplans in das Standardprogramm aufgenommen. Dieser wird ohnehin auch auf Wunsch seitens der Patient:innen, für Studien und Diäten in anderen Fällen benötigt und gerne angenommen.

### Technische Voraussetzungen und Protokoll

Für die Durchführung einer kardialen PET/MRT sind wichtig: EKG-Gating der PET in der Enddiastole, eine PET-Bewegungskorrektur sowie eine Software zur Fusion im Postprocessing, um eine hohe Qualität der PET und der Fusion zu erhalten [1]. Falls keine integrierte (hybride) PET/MRT oder alternativ PET/CT und MRT vorhanden sind, ist auch eine nachträgliche Fusion oftmals möglich, wenngleich die exakteste Übereinstimmung naturgemäß mit einem integrierten PET/MRT-Scanner zu erzielen sein wird.

Für eine vollständige kardiale PET/MRT-Untersuchung ist folgendes Protokoll zu empfehlen: Nach [^18^F]FDG-Applikation folgt eine Stunde Ruhezeit im Liegen, um ein Uptake in die Zielzellen und Clearing des Tracers aus dem Blutpool zu erzielen. In der PET/MRT-Untersuchung selbst folgen auf die Schwächungskorrektur die diagnostischen Herz-MRT-Sequenzen: T1- und T2-Mapping, funktionelle Cine-Sequenzen, Late-Gadolinium-Enhancement (LGE) in 2D und 3D (zur Fusion mit dem PET) sowie zum Abschluss T1-Mapping nach Kontrastmittelgabe.

### Messwerte PET/MRT

#### Late-Gadolinium-Enhancement

Sinnvoll ist es, die LGE-Bilder zu Beginn der Analyse auszuwerten, um etwaige ischämische oder nichtischämische Narben zu erkennen. Im Folgenden werden dann die übrigen MRT-Bilder wie Mapping und Funktion sowie die PET-Aufnahmen ausgewertet. Eine Quantifizierung des LGE (in Prozent der linksventrikulären Myokardmasse) hat zwar prognostischen Wert [13, 14], im klinischen Kontext ist diese Angabe bislang noch kein Standard.

#### Mapping

T1- und T2-Mapping sowie ECV-Mapping („extracellular volume mapping“) werden quantitativ ausgewertet, deshalb sollte bei der Auswertung besonderes Augenmerk möglichen Störgrößen wie Artefakten, Bewegung und Partialvolumen durch angrenzendes Blut und epikardiales Fett gelten. Eine lokale Kontrollgruppe mit gesunden Probanden oder publizierte Normalwerte und Obergrenzen müssen auf den Scanner- und Sequenztyp zugeschnitten sein und sind nicht übertragbar [15]. T1- und ECV-Mapping zeigen quantitativ eine Myokardschädigung an, welche dann mithilfe von T2-Mapping in ödematös-entzündliche bzw. fibrotische Veränderungen unterschieden werden kann.

#### Funktion

Die Funktionsauswertung in den Schichten der kurzen Achse gilt als Referenzstandard und kann in der Regel (semi)automatisiert erfolgen, sollte jedoch immer visuell Schicht für Schicht kontrolliert werden, um die hohe Qualität zu erhalten [15] und etwaige Fehlmessungen zu korrigieren. Insbesondere die unterschiedliche Höhe der basalsten Schicht in Diastole und Systole durch die longitudinale Kontraktion werden durch unerfahrene Auswerter und automatisierte Programme häufig falsch erkannt, was zu einer falsch-niedrigen Ejektionsfraktion führen kann [16].

#### Positronen-Emissions-Tomographie

Die myokardialen Verteilungsmuster des FDG Uptake werden folgendermaßen eingeteilt [11, 17–20]:

1. vollständige Suppression (keine Aufnahme),
2. fokal,
3. fokal auf diffus
4. diffus.

Bei diffuser myokardialer Aufnahme ist eine seltene ubiquitäre diffuse Inflammation von einer häufigeren insuffizienten myokardialen FDG-Suppression zu unterscheiden. Um falsch-positive PET-Befunde aufgrund inadäquater myokardialer Suppression zu vermeiden, wird folgender Ansatz verwendet: Die PET/MRT gilt nur als positiv, wenn in der MRT eine myokardiale Abnormalität (positives T1/T2-Mapping und/oder LGE) vorliegt; Tab. 1 [11, 12].

**Table 1:** 

Befund im PET/MRT	Wertung
MR−/PET−	Keine Inflammation
MR+/PET+	Floride, aktive Inflammation
MR+/PET−	Fibrosierung, keine Inflammation
MR−/PET+	Falsch-positive PET

Für die Auswertung der PET bieten sich neben dem standardisierten Uptake-Wert (SUV) für die Einschätzung der Inflammation die folgenden Ratios an [11]:

##### TBRmax.

„Maximum (normal myocardial) tissue-to-background ratio“, die Ratio aus normalem Myokard zum Blutpool Hintergrund. Diese dient als Überprüfung der Aktivität im normalen Myokard und der korrekten myokardialen Suppression. Werte zwischen ca. 1,1 und 5 sind hier zu erwarten, deutlich höhere Werte können zwar bei starker diffuser Inflammation auftreten, sprechen aber eher für eine insuffiziente myokardiale Suppression.

##### TNMRmax.

„Maximum target-to-normal myocardium ratio“, die Ratio von inflammatorischem zu normalem Myokard ist ein Maß für die Inflammation. Hier ist ein Wertebereich zwischen ca. 1,5–2,5 zu erwarten, somit teilweise deutlich weniger als in soliden Tumoren oder abszedierenden inflammatorischen Prozessen in der PET. Als Kritikpunkt kann dieser Wert bei hoher diffuser Inflammation niedrig bleiben und nicht zwingend die absolute Inflammation messen (Tab. 2).

**Table 2:** 

Ratio	Messwerte SUV („Region oder volume of interest“, ROI/VOI)
*TBRmax* „Maximum (normal myocardial) tissue-to-background ratio“	Normales Myokard/Blutpool (Hintergrund)
*TNMRmax* „Maximum target-to-normal myocardium ratio“	Inflammatorisches Myokard/normales Myokard

## Anwendung bei inflammatorischer Herzerkrankung

### Sarkoidose

Die Sarkoidose ist eine systemische granulomatöse Erkrankung und kann sämtliche Organsysteme betreffen. Patienten mit kardialer Beteiligung der Sarkoidose können sich klinisch ganz unterschiedlich präsentieren: von völligem Wohlbefinden über Herzinsuffizienz, Rhythmusstörungen bis hin zum plötzlichen Herztod [13, 21, 22]. Die granulomatöse myokardiale Inflammation ist initial potenziell reversibel, kann jedoch auf Dauer in eine irreversible Fibrosierung übergehen [12].

**Figure 2:**
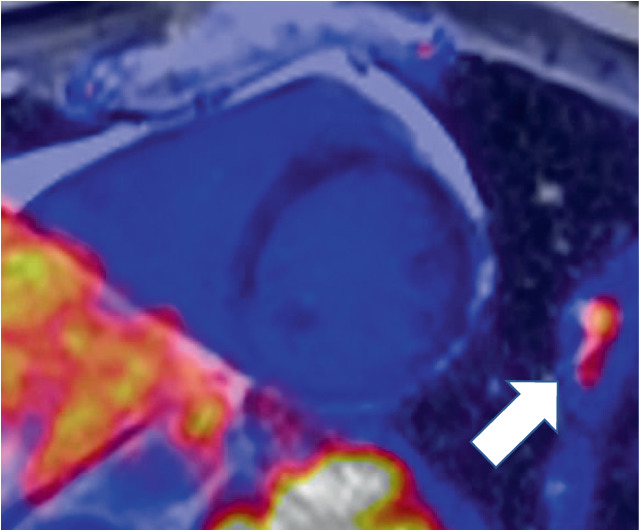

Die kardiale PET/MRT kann hier ihre Stärken ausspielen: Erkennen einer myokardialen Beteiligung, Charakterisierung der Veränderungen in aktive oder chronische Prozesse (oder ggf. beides), und weiteres Monitoring der kardialen Sarkoidose [12]. So lassen sich frühzeitig therapiebedürftige, potenziell noch reversible Stadien der kardialen Sarkoidose erkennen und weiter unterteilen [12]; Abb. 2, 3 und 4.

**Figure 3:**
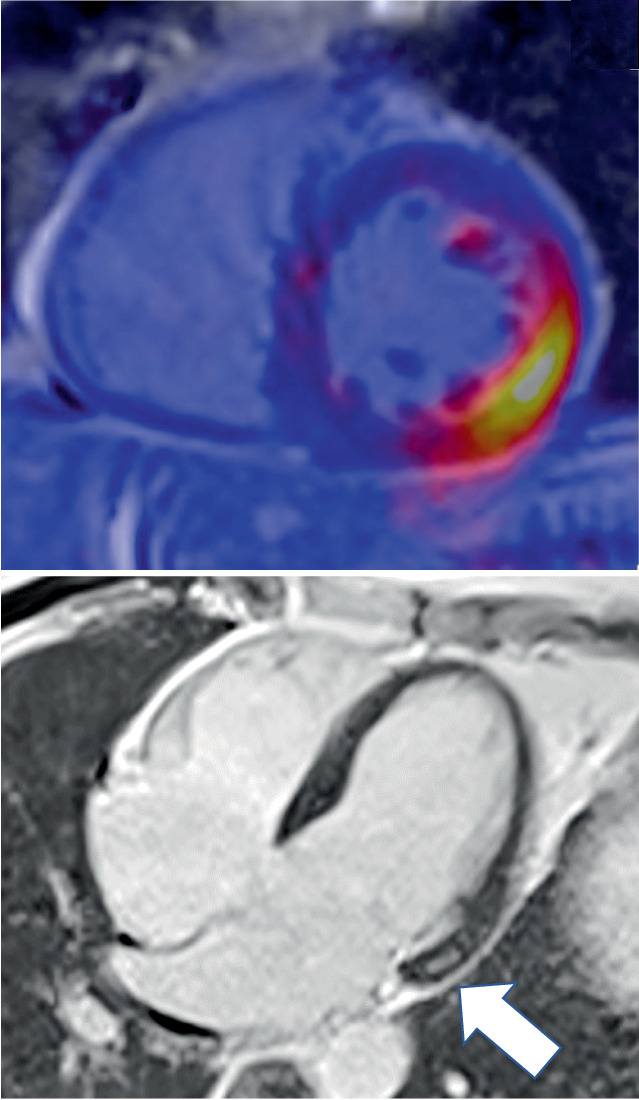

**Figure 4:**
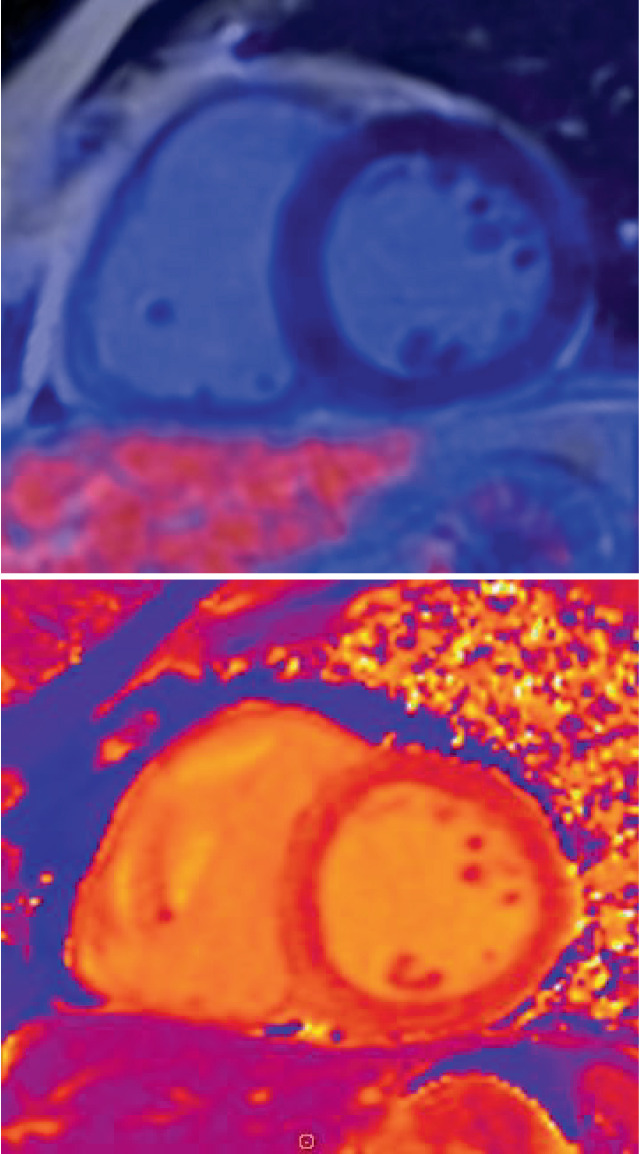

Mit der PET kann die notwendige immunsuppressive Therapie auch im Verlauf untersucht und deren Erfolg kontrolliert werden [23]. Sowohl das mittels MRT bestimmte LGE als auch die PET/MRT erlauben in dem Zusammenhang auch prognostische Aussagen [13, 24]. Das Vorhandensein von LGE ist mit dem Auftreten unerwünschter Ereignisse korreliert [13]. Auf der anderen Seite bietet ein negatives LGE einen hohen negativen prädiktiven Wert für unerwünschte Ereignisse, so dass in vielen Zentren eine kardiale MRT bei Patient:innen mit Sarkoidose routinemäßig durchgeführt wird, da die Symptome der Patient:innen sowie EKG und Echokardiographie häufig nicht wegweisend für eine kardiale Beteiligung im Rahmen einer Sarkoidose sind.

### Myokarditis

Die Myokarditis ist aufgrund ihrer unterschiedlichen Phänotypen und histologischen Stadien (akut, subakut, chronisch, abgeheilt) und einem möglichen Übergang zu einer dilatativen Kardiomyopathie häufig ein diagnostisch schwer zu fassendes Chamäleon [21, 25, 26]. Die endomyokardiale Biopsie gilt weiterhin als Referenzstandard, wenngleich nach Nutzen-Risiko-Abwägung nicht jeder Myokarditis Verdacht mittels Biopsie abgeklärt werden kann und muss [27]. Myokardiale Narbenbildung (insbesondere anteroseptal im LGE) ist mit einer erhöhten Mortalität vergesellschaftet, so dass diese Patient:innen nach Risikostratifizierung durch die kardiale MRT einen höheren Bedarf für eine umfassende kardiologische Nachsorge haben [14, 28–30].

Nur ein umfassendes MRT-Protokoll im Kontext mit Klinik und Laber erlaubt eine gute ausreichende Diagnostik und Differenzierung zwischen den einzelnen Stadien der Myokarditis [31]. Die akute und subakute Myokarditis werden durch die aktuellen Lake-Louise-Konsensusempfehlungen diagnostiziert: Erforderlich zur Diagnose ist ein Ödem in der T2-Bildgebung (konventionelle T2-Wichtung oder T2-Mapping) und zusätzlich der Nachweis einer myokardialen Schädigung in der T1-Bildgebung (T1-Mapping, ECV-Mapping, LGE; [32]).

Das T2-Mapping kann insbesondere bei der Detektion einer ggf. persistierenden ödematösen Inflammation bei subakuter oder chronischer Myokarditis helfen [33]. Die PET in der kardialen Hybrid-PET/MRT dient als Referenz für die Darstellung einer möglichen Inflammation [34]. Allerdings fehlt es bislang an systematischen und prospektiven Studien [35, 36], um hier eine klare Empfehlung auszugeben. Ob die MRT-Technik des T2-Mappings eventuell irgendwann die PET ersetzen kann, wird sich in zukünftigen großangelegten Studien zeigen. Aktuell muss sich das T2-Mapping am Referenzstandard PET für die Detektion einer myokardialen Inflammation messen lassen.

## Fazit für die Praxis

- Die Diagnostik einer inflammatorischen Herzerkrankung sowie deren Charakterisierung in akut vs. chronische Prozesse gelingt mit der kardialen Hybrid-Positronen-Emissions-Tomographie/Magnetresonanztomographie (PET/MRT), wie am Beispiel der kardialen Sarkoidose gezeigt werden konnte.
- Weitere Studien sind notwendig, um die Rolle der kardialen PET/MRT bei inflammatorischer Kardiomyopathie nicht nur bezüglich Diagnose, sondern auch bezüglich des weiteren klinischen Verlaufs (Monitoring, ggf. Therapieanpassung) und der Prognose dieser Patient:innen zu untersuchen.
